# The Anti-Inflammatory Activities of Fermented Curcuma That Contains Butyrate Mitigate DSS-Induced Colitis in Mice

**DOI:** 10.3390/molecules27154745

**Published:** 2022-07-25

**Authors:** Al Borhan Bayazid, Soo Ah Jeong, Chae Won Park, Da Hee Kim, Beong Ou Lim

**Affiliations:** Department of Applied Life Science, Graduate School of Konkuk University, Chungju 27478, Korea; bayazid92@diu.edu.bd (A.B.B.); jje318@kku.ac.kr (S.A.J.); woni109@kku.ac.kr (C.W.P.); dahee9709@naver.com (D.H.K.)

**Keywords:** inflammations, colitis, butyrate, Curcuma, *Clostridium butyricum*

## Abstract

Inflammatory bowel disease is characterized by a radical imbalance of inflammatory signaling pathways in the gastrointestinal tract, and it is categorized into two diseases, such as Crohn’s disease and ulcerative colitis. In this study, we investigated anti-inflammatory activities using fermented Curcuma that contains butyrate (FB). Nitric oxide production in RAW 264.7 cells and the expression of inducible nitric oxide synthase in the intestinal mucosa appears to be enhanced in active ulcerative colitis. Here, the cytotoxicity, physiological activity, and anti-inflammatory efficacy of FB in colitis animals were investigated. To verify the anti-inflammatory effect, this study was conducted using the dextran sulfate sodium (DSS)-induced colitis mice model. As a result, non-toxicity was confirmed, and anti-inflammatory effects were revealed by inducing a reduction of LPS-induced NO production. In the DSS-induced colitis, reduced weight was recovered and a decrease in inflammatory factors Ig-E and TNF-α in the mesenteric lymph node (MLN) and spleen was induced, and it was confirmed to help with the morphological remodeling of the intestine. In conclusion, this paper suggests that FB can help to alleviate intestinal inflammation and to improve the intestinal environment, with the help of morphological remodeling.

## 1. Introduction

Inflammatory bowel disease (IBD) is a multi-factory chronic disease, typically a gastrointestinal inflammatory disease caused by changes in the composition and the function of intestinal microbes and various genetic and environmental factors [1]. It has been said that abnormalities in the intestinal microbiome are increasingly considered to be related to IBD and strongly influenced by the components of Western lifestyles. Bacteria that ferment fiber and produce short-chain fatty acids (SCFAs) typically decrease mucous membranes and feces in IBD patients compared to healthy people [2]. IBD can be categorized as Crohn’s disease (CD) that may occur in any part of the gastrointestinal tract and ulcerative colitis (UC) onset in the colon.

UC models have been developed to study pathogenic studies and genes, such as gene knockout (KO), interleukin (IL)-2 receptor-alpha, IL-10, T cell receptor, and tumor necrosis factor (TNF)-α untranslated domain [3,4,5]. UC induced by heparin-like polysaccharides, such as dextran sulfate sodium (DSS), is simple, and it has the quality and the uniform form of the lesion in the colon. DSS is commonly used to induce the formation of mouse colitis, mimicking clinical and histologic features with UC characteristics by first interfering with the intestinal barrier function between 3 and 7 days and then stimulating local inflammation. In particular, the expression of pro-inflammatory cytokine and chemokine (IL-1, IL-6, TNF-α, Interferon-γ) upregulates [6,7,8], whereas the synthesis of an anti-inflammatory cytokine such as IL-10 downregulates [9]. Other parameters, such as weight loss and colon length reduction, rising bone marrow oxidase levels (suggesting neutrophil infiltration into epithelium), and high histologic and endoscopic scores, characterize colitis in mice [10]. DSS caused colitis, but mechanisms of action are still unknown. However, prior studies have shown that DSS competes with poly to induce biological mechanism collapse (such as inhibitory effects on reverse transcriptase activities affecting key cell functions), and dextran sulfate inhibits ribonuclease action [11].

SCFAs, such as acetate, propionate, and butyrate, are important metabolites for maintaining intestinal homeostasis. In particular, the gut of colitis patients is reported to induce a reduction in the production of SCFAs by an important immune-regulating molecule, primarily butyrate, the gut microbiota [12]. Among them, butyrate plays an important role in protecting intestinal homeostasis, which acts on both adaptive and congenital immunity. Curcuma is native to southeast Asia, Bangladesh, India, Pakistan, and Australia, and it has been reported for several bio-functional activities [13]. *Clostridium butyricum* bacterium has been widely used to produce butyrate through the fermentation of herbs [14,15]. Butyrate is a fatty acid composed of six carbon atoms, which is a major source of energy for intestinal microbial fermentation, colorectal cells, and a very important ingredient for intestinal health [2,16]. SCFA absorption is facilitated by substrate transporters, such as monocarboxylate transporter 1 (MCT1) and Sodium-coupled monocarboxylate transporter 1 (SMCT1), to promote cell metabolism. Its absorption is facilitated by substrate transporters, such as MCT1 and SMCT1, to promote cellular metabolism [17]. In addition, SCFAs can activate the signal activity that controls the immune function by sending signals through cell-surface G protein-coupled receptors (GPRs), such as GPR41, GPR43, and GPR109A. Genetic mouse models support the main role of these GPRs in regulating bowel inflammation [18]. Previous clinical studies have reported that SCFA is generated in large quantities by intestinal microbiota, reaching concentrations of approximately 13 ± 6 mmol/kg at the end and approximately 80 ± 11 mmol/kg at the descending colon. In the intestine, butyrate can be synthesized through four pathways: acetyl-CoA, glutamate, lysine, and succinate [19]. Butyrate can regulate the activation of regulatory T cells, increasing the acetylation of histones and decreasing the activation of nuclear factor kappa B (NF-κB) [20]. In addition, it can produce mucus from epithelial cells and relocate dense bonded proteins. We investigated the treatment potential of SCFA for colitis by stimulating SCFA-generated bacteria through direct application or prior probiotic approaches.

## 2. Materials and Methods

### 2.1. Sample Preparation

The FB sample was kindly provided by BINOTECH, Daegu, Korea. In this study, Curcuma was fermented using *Clostridium butyricum*, which produces butyrate. Curcuma was fermented for 3 days in an incubator at 37 °C, using *Clostridium butyricum* strain, and the medium was cultured using RCM difco. After filtering, the lyophilized powder of FB was stored at 4 °C before use.

### 2.2. Chemicals and Reagents

3-(4,5-dimethylthiazol-2-yl)-2,5diphenyltetrazolium bromide) (MTT), DMSO (dimethyl sulfoxide), sodium nitrite, 1,1-diphenyl-2-picrylhydrazyl (DPPH), sodium butyrate (NaB), and sulfasalazine were purchased from Sigma Aldrich (Saint Louis, MO, USA). DMEM (Dulbecco’s Modified Eagle’s Medium), DPBS (Dulbecco’s phosphate-buffered saline), FBS (fetal bovine serum) penicillin and streptomycin (P.S), Trypsin EDTA were purchased from WELGENE Inc. (Seoul, Korea). All the other chemicals and reagents were analytical grade and used without any further purification. 

### 2.3. DPPH Radical Scavenging Activities

DPPH is a synthetic free radical that has been widely used for investigating abiotic antioxidant activities by neutralizing it [7]. A total of 80 μL of 2 mM DPPH was poured into a 96-well plate, and several concentrations of FB (80 μL) were added to the 96-well plate and then incubated at room temperature (25 °C) in the dark for 30 min. Then, absorbance was taken at 517 nm in a 96-well plate reader (VersaMax, Molecular Device, San Jose, CA, USA).

[DPPH radical scavenging activity (%) = {(C − D) − (A − B)}/(C − D) × 100]

where C is the absorbance of DPPH + solvent, D is the absorbance of solvent + methanol, A is the absorbance of sample or standard + DPPH, and B is the absorbance of sample or standard + methanol.

### 2.4. Cell Viability 

Murine macrophage RAW 264.7 cell line were purchased from the Korean Cell Line Bank (Seoul. Korea) and maintained in DMEM with 10% FBS and 1% P.S in a cell incubator. Cells were cultured in an incubator at 37 °C with 5% CO_2_ and 95% humidified conditions. Cell viability was measured by MTT assay. The RAW 264.7 cells were seeded in a 96-well plate as 5 × 10^4^ cells/well and incubated for 24 h. Then, the cells were treated with different FB concentrations (10–1000 μg/mL) and incubated for another 24 h. Afterward, an MTT of 0.5 mg/mL at the final concentration was added and kept for 2 h at 37 °C, then the formed formazan blue was dissolved in DMSO and measured in a 96-well plate reader (VersaMax, Molecular Device, San Jose, CA, USA) at 570 nm. The control group was considered 100%.

### 2.5. Determination of Nitric Oxide (NO) Production

The RAW 264.7 cells were plated in a 96-well plate at a density of 5 × 10^4^ cells/well and incubated for 24 h. RAW 264.7 cells were pretreated with different FB concentrations (10–1000 μg/mL) for 1 h before incubating with LPS (100 μg/mL) for 24 h. Nitric oxide (NO) production was determined by the reaction of a macrophages culture supernatant with a Griess reagent (100 μL, 1% sulfanilamide, 0.1% *N*-1-naphthyl ethylenediamine). The culture supernatant (100 μL) was mixed with the Griess reagent (100 μL) at 25 °C and shaken gently for approximately 10 min. Finally, a microplate reader was used to measure the absorbance of the mixture at 540 nm.

### 2.6. Animals

Five-week-old male BALB/c mice were purchased from DBL (Chungcheongbuk-do, Korea). The initial body weight of the mice was 30 ± 2 g, and mice were acclimatized for 1 week in an SPF animal room under controlled conditions, where the temperature was 25 ± 2 °C, humidity was 55–65%, a lighting regimen of 12-h light/12-h dark and adequate water and a standard diet were supplied. After 1 week of acclimatization, the mice were randomly divided into 6 groups; (1) control group was fed a normal diet, (2) DSS (3% DSS in water), (3) NaB (Sigma, Saint Louis, MO, USA) 22 mg/kg BW, (4) FB (low) 50 mg/kg BW, (5) FB (high) 100 mg/kg BW, and (6) Sulfasalazine (positive control) 50 mg/kg BW. All groups had received 3% DSS in drinking water for 10 days except the control group. Then, the DSS group received a normal diet only, and the NaB (Sigma), FB (low), FB (high), and Sulfasalazine groups were treated through oral administration for 14 days besides the normal diet. Sulfasalazine is considered the positive control group. Animal experiments were performed using a protocol according to the National Institutes of Health guidelines and approved by the Institutional Animal Care and Use Committee (IACUC) of Konkuk University (approval number: KU20227).

### 2.7. Measurement of Body Weight and Gut Length

After DSS-induced for 10 days, then FB, NaB, and Sulfasalazine were treated for 14 days. The body weight of mice was weighed on a measuring scale. 

The mice were decapitated after treatment and organ specimens had been collected. Afterward, the gut length was measured by bar scale. The gut length was expressed in centimeters (cm).

### 2.8. Determination of TNF-α

The content in spleen and mesenteric lymph node (MLN) tissue of pro-inflammatory cytokine, TNF-α, was determined according to the enzyme-linked immunosorbent assay (ELISA) kit (mouse ELISA, R&D Systems, Minneapolis, MN, USA), following the manufacturer’s instructions. Briefly, to prepare the plate, the captured antibody was diluted to a working concentration and used to coat a 96-well plate with 100 μL per well. Plates were sealed with parafilm and incubated overnight at 25 °C. The detected antibody was aspirated and washed three times with phosphate-buffered saline (PBS)-Tween (T). After blocking with 2% bovine serum albumin (BSA), the ELISA plate was aspirated and washed three times. Each 100 µL sample was added to each well and incubated at 25 °C for 2 h. After incubation, the suction step was repeated. Wells were then filled with 100 µL diluted detection antibody and incubated at 25 °C for 2 h. After aspiration, 100 µL streptavidin-HRP diluted solution (1:20) was added to each well, incubated in the dark at 25 °C for 20 min and then aspirated. A substrate solution (100 μL) was added and stored in the dark as before (6–12 min) until color development. Next, a 50 µL stop solution was added to each well, and the absorbance at 450 nm was measured.

### 2.9. Serum IgE Analysis

Blood serum was collected from the mice blood sample by centrifugation at 2000× *g* for 10 min at 4 °C. The serum levels of cytokines (pg/mL) were quantified using ELISA commercial kits of IgE, following the manufacturer’s instructions (R&D Systems, Minneapolis, MN, USA).

### 2.10. Hematoxylin and Eosin (H&E) Staining of Colon Tissue

The colon was collected from mice and fixed with 4% paraformaldehyde. After fixation, large intestines were embedded in paraffin and cut into slices 5 μm thick. Then, the morphology of colon tissue was stained with H&E, and images were taken in a microscope.

### 2.11. Statistical Analysis

The results are presented as the means ± SEM for all experimental data. Data were analyzed with Microsoft Excel 2016 and GraphPad Prism 5.0 software (GraphPad Software, Inc., San Diego, CA, USA) using one-way analysis of variance and non-parametric according to paired Student’s *t*-tests were used to test for significance. * *p* values < 0.05 were deemed to be statistically significant.

## 3. Results

### 3.1. Gas Chromatography and Mass Spectrometry (GC/MS) of FB 

FB was provided from BINOTEC in Daegu, Korea. It was analyzed through GC/MS to determine the butyrate content and the experiment was done by K-BIO (Konkuk University, Chungju, Korea). FB was analyzed, respectively, and the results contained 0.19 g of butyrate per 100 g of lyophilized FB (Table 1).

### 3.2. Antioxidant and Anti-Inflammatory Activities of FB and the Effect on Cell Viability

**Figure 1:**
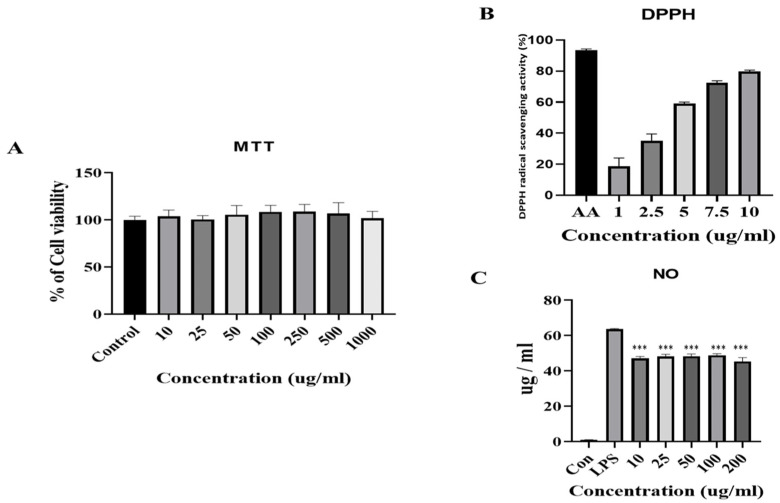
Biological effects of FB in RAW 264.7 cells. (**A**) Changes of the cell viability of RAW 264.7 cell by MTT. (**B**) DPPH radical scavenging activity of butylate. (**C**) Inhibition of NO product by butylate. Data are represented as mean ± SD (*n* = 4), analyzed by one-way ANOVA, followed by the Dunnett’s test. *** *p* < 0.01 vs. LPS treated group.

To investigate the bio-functional activities of FB, we measured the cell viability, anti-inflammatory efficacy in RAW 267.4 cells, and antioxidant efficacy through DPPH radical scavenging activities. First, cell viability was performed by MTT assay in the RAW 267.4 cells. There was no cytotoxicity up to 1000 μg/mL of FB (Figure 1A). Next, a chemical experiment, DPPH, was conducted to find out the antioxidant ability, and a strong antioxidant ascorbic acid was used as a comparative group. FB showed antioxidant ability from a concentration of 1 μg/mL (about 20%), and it showed an increase in a concentration-dependent manner (Figure 1B). These results showed that low concentrations of fermented products have strong antioxidant properties. As for the anti-inflammatory effect, LPS (1 μg/mL) was treated on macrophages to induce the secretion of NO, and when the FB was treated for each concentration (from 10 μg/mL to 200 μg/mL), the generation of NO was investigated through Griess assay (Figure 1C). Through the results of the physiologically active experiment, this study showed that FB is a non-toxic, antioxidant, and anti-inflammatory substance.

### 3.3. The Effect of FB in DSS-Induced Colitis Mice

**Figure 2:**
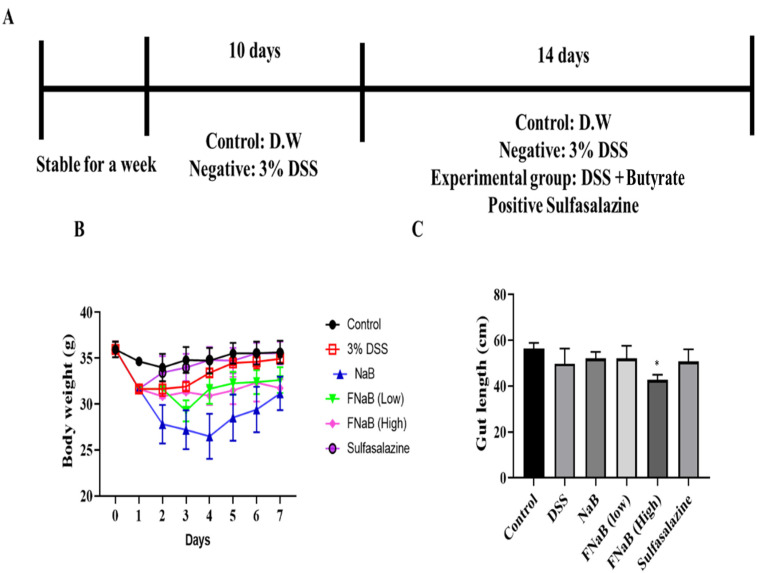
Animal modeling and morphological analysis of FB in DSS-induced colitis mice. (**A**) Schedule of DSS-induced colitis modeling. (**B**) Body weight change of DSS-induced mice. (**C**) Changes in intestinal length in DSS-induced colitis. The histograms were presented as Mean ± SEM, where *n* = 5. and *p*-Value less than 0.05 was expressed as statistically significant. * *p* < 0.05 compared to the DSS group.

**Figure 3:**
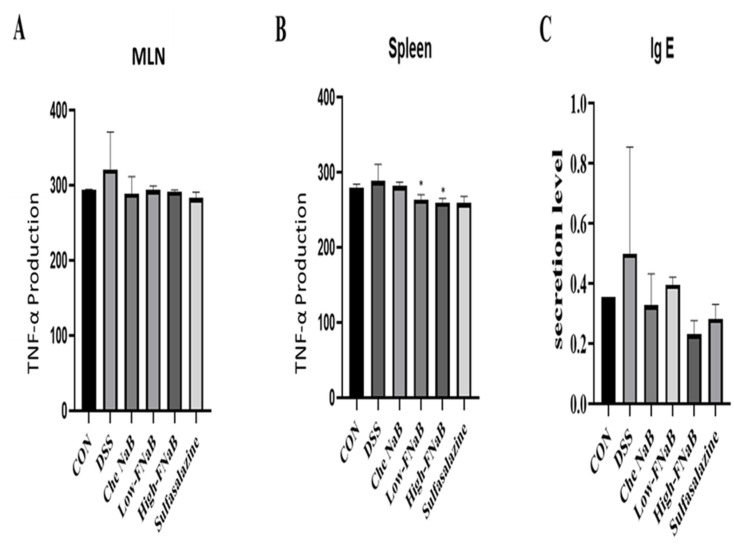
Pro-inflammatory cytokine inhibitory effects of FB. (**A**,**B**) Production of TNF-α by DSS in mesenteric limp node and spleen. (**C**) The production of Ig-E in serum. The histograms were presented as Mean ± SEM, where *n* = 5 and *p*-Value less than 0.05 was expressed as statistically significant. * *p* < 0.05 compared to the DSS group.

We studied the physiological activity results of FB and examined the anti-inflammatory efficacy in DSS-induced colitis mice to further verify anti-inflammatory efficacy. The colitis mice were derived using 3% DSS (Figure 2A): DSS was dissolved in drinking water (D.W) and they were fed for 10 days, gradually decreased body weight (Figure 2B), and the animals with severe colitis died. Next–except for the control group and the DSS negative group–animals received an oral administration of NaB (from Sigma-Aldrich, Saint Louis, MO, USA), a low concentration and a high concentration of FB, and sulfasalazine (positive control, 50 mg/mL) every day. Weight was measured daily, and after completion of administration, intestines, serum, spleen, and MNL were sampled to evaluate the efficacy of the FB. Weight was recovered in groups that received the oral administration (Figure 3B). The length of the intestine was reduced due to DSS (Figure 2C). Perhaps, NaB and FB do not affect the length of the colon.

### 3.4. TNF-α and IgE Release in DSS-Induced Colitis Mice

The DSS-induced colitis model is known to cause inflammation only in the large intestine. We isolated MLN and spleen from colitis mice and quantified the secretion of cytokine TNF-α. The TNF-α level was measured using an ELISA kit and, in the MLN and in the spleen, TNF-α level increased in the DSS group, decreased only in MNL in animals that ate NaB, and decreased in both the FB groups (low and high group) and the positive groups. Finally, the immune factor Ig-E level was measured. Serum was separated from the blood of each animal and measured (Figure 3C). The Ig-E level was increased in animals induced colitis with DSS, and the IgE level was decreased in NaB, FB, and positive control groups. These results suggested that FB shows anti-inflammatory efficacy in the intestines of animals with colitis, and further decreases in TNF-α and IgE also affect systemic immunity.

### 3.5. Histology of Large Intestine in DSS-Induced Colitis Mice

**Figure 4:**
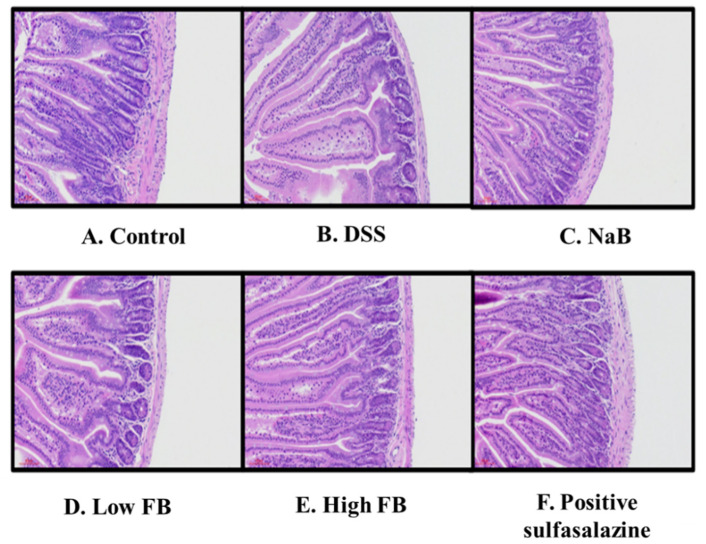
Morphological analysis of the large intestine of FB in colitis mice. All animals were administered oral during the recovery period. (**A**) Control (**B**) DSS-induced model (**C**) Sodium butyrate (Sigma-Aldrich), (**D**), Low concentration (50 mg/kg) of FB. (**E**), High concentration (100 mg/kg) of FB. (**F**), Positive control (sulfasalazine, 50 mg/kg).

Finally, we observed morphological changes in the large intestine tissues of DSS-induced colitis mice (Figure 4). The lengths of the epidermis and the dermis were significantly reduced in the large intestine of DSS-induced colitis mice (Figure 4B). On the other hand, a morphologically significant recovery was observed in the group fed NaB (Figure 4C). However, a low-concentration intake in FB administered mice also showed morphological recovery (Figure 4D), and many recoveries were confirmed in animals treated with high-concentration FB (Figure 4E). In these results, FB contributes to the morphological recovery of the large intestine but not a full recovery. This result showed similar results to the relationship between the length of the field in Figure 2C.

## 4. Discussion

In this study, butyrate, a metabolite of intestinal microorganisms, was produced through fermentation by bacteria to find the effect of relieving inflammation using macrophages and colitis in mice. As a result, concentration-dependent strong antioxidant and anti-inflammatory effects were found in macrophages, and cytokine production and the inflammatory factor decreased in DSS-induced mice.

Colitis is induced by a radical imbalance in the activation of pro-inflammatory and anti-inflammatory signaling pathways in the colon. NF-κB is a hallmark regulator of gene transcription controlled by translocation of NF-κB into the nucleus. NF-κB signaling is often dysregulated, resulting in inflammation in colitis patients [21]. Cytokines not only drive intestinal inflammation and diarrhea in colitis but may also regulate extra-intestinal disease manifestations (for example, arthralgia or arthritis) and systemic effects. Furthermore, cytokines seem to have a crucial role in driving complications of IBD, such as intestinal stenosis, fistula formation, and colitis-associated neoplasias [22]. Anti-cytokine therapies involving TNF-specific agents form a pivotal factor in clinical therapy in both CD and UC. TNF-specific antibodies suppress chronic intestinal inflammation, and they may induce mucosal healing in colitis [23]. Chronic inflammation contributes to a high risk of colorectal cancer (CRC) development. TNF-α and Ig-E are cytokines and important inflammatory mediators that play a pivotal role in malignant cellular proliferation, angiogenesis, and tissue invasion and metastasis [22]. In this study, we tested that butyrate down-regulated the levels of pro-inflammatory factors TNF-α and Ig-E in a dose-dependent manner.

Industrial development is creating many harmful substances that cause inflammation in the human body. For that reason, many people are increasingly concerned about their health, and they prefer healthy functional foods that can defend against harmful substances [24,25]. Red ginseng is widely known as a material for enhancing immunity. However, researchers are discovering many other materials through research. Most of the materials with anti-inflammatory effects contain significant numbers of polyphenols and various anthocyanins [26]. In addition, research results show that materials that are in the spotlight show the beneficial effects on the human body, such as skin and brain health, of materials that help intestinal health [27]. Changes in the intestinal environment can be induced through various beneficial bacteria (such as Bifidobacterium, Lactobacillus, Lactococcus, and Enterococcus) but it is known that microorganisms in the intestine can also be changed into metabolites that eat and discharge nutrients [28]. We showed the results of anti-inflammatory efficacy and inflammation relief in colitis using FB among metabolites. Additionally, we have also recently derived the results that FB relieves inflammation in the brain [4,29]. In these results, it is hypothesized that FB showed an overall anti-inflammatory effect on the DSS-induced mice and on RAW 264.7 cells. 

In conclusion, through this study, FB showed non-toxic, anti-inflammatory, and strong antioxidant effects through macrophages, resulting in alleviation of inflammation in the DSS-induced mice model. However, FB showed morphological remodeling of the large intestine, but it was not completely recovered. FB showed results that could alleviate inflammation of the whole body; and, through this material, we envisage that it will be a material for anti-inflammatory health functional foods and for probiotics and co-effects; further research is needed.

## Figures and Tables

**Table 1:** GC/MS of Fermented sample.

*Name*	*Content*
*Butyrate*	0.19 g per 100 g

## Data Availability

All data and the information about materials and methods in this study are available from the corresponding author on request.

## Sample Availability Statement

Samples of the compounds are available from the authors.
